# The influence of different conflict management styles on depressive symptoms in employees: the mediating role of emotional exhaustion

**DOI:** 10.3389/fpubh.2024.1407220

**Published:** 2024-10-07

**Authors:** Qihan Zhang, Yueran Lin, Yimou Zhang, Shaofeng Yang

**Affiliations:** ^1^Department of Psychology, Tianjin Normal University, Tianjin, China; ^2^Academy of Psychology and Behavior, Tianjin Normal University, Tianjin, China; ^3^Xiqing District Federation of Trade Unions, Tianjin, China; ^4^School of Psychology, Inner Mongolia Normal University, Hohhot, China

**Keywords:** coordination style, concession style, emotional exhaustion, depression, employee

## Abstract

Although some studies have found that conflict management styles impact employees' levels of depression, the expressions of employees' depressive symptoms under various conflict management styles and the underlying mechanisms remain to be elucidated. In this study, a total of 1,312 employees were gathered through an online survey to examine their current depressive status. Drawing on the conservation of resources theory, the mediating role of emotional exhaustion in the association between conflict management style and employee depression was further explored. The findings revealed that the prevalence of moderate to severe depression among the workers was 13.9%. Employees with different conflict management styles displayed distinct depression scores. The high coordination style group exhibited a significantly lower depression score compared to the high concession style group. Emotional exhaustion played a mediating role in the impact of both the coordination style and the concession style on employees' depression. It can be seen that the mental health needs of the workplace are imminent. The intervention measures to improve the mental health of employees in the working environment should take into account the cultivation of the coordination style of employee conflict management, reduce their emotional exhaustion, and stay away from depression through the acquisition of constructive and positive coping styles.

## 1 Introduction

According to the International Labor Organization (ILO), the global workforce constitutes ~60% of the world's population (1). Findings from the ninth national survey on the status of Chinese workers conducted by the Federation of Trade Unions indicate that as of February 2023, there are around 402 million workers in China (2). This highlights the substantial size of the workforce, underscoring its crucial role in global economic advancement and social stability. Statistics from the World Health Organization (WHO) show that in 2019, about 15% of workers worldwide grappled with mental health disorders (3). A national occupational health literacy monitoring statistical survey organized by China's National Health Commission in 2022 revealed that 15% of monitored employees exhibited depression, anxiety, and other adverse emotions (4). Currently, mental health issues have emerged as a leading cause of disability (5), with depression standing out as one of the most prevalent mental disorders (6). The American Psychiatric Association estimates that 1 in 15 adults suffers from depression, and ~1 in 6 individuals will experience depression at some point in their lives (7). Employee depression significantly impacts job performance, productivity, absenteeism, and disability costs (8). The annual cost of absenteeism or reduced productivity in the United States is conservatively estimated to exceed $300 billion (9). Moreover, worker depression escalates medical expenses, medication costs, family burdens, and more, emphasizing the extensive repercussions of employee depression that warrant broad societal attention. China has implemented various policies and regulations such as the “14th Five-Year National Health Plan” (80), and “22 Departments on strengthening mental health services” (78), which mandating the comprehensive advancement of occupational mental health services.

Workplace conflict is typically defined as discord between colleagues or between leaders and employees stemming from persistent or unresolved actual (or perceived) disparities, encompassing conflicts triggered by organizational factors like resource scarcity and ineffective communication, interpersonal factors such as divergent values and significant personality differences, and conflicts originating from work tasks themselves (10). Encountering these conflicts daily in the actual workplace has emerged as a prominent stressor, exerting a substantial negative impact on the physical and mental wellbeing of employees (11–13). However, studies have failed to establish a link between workplace conflict and depressive symptoms. In their investigation into the association between depression symptoms and workplace conflict among a sample of 2,164 workers in 65 different occupations, Zuelke et al. (14) discovered no correlation between the two variables. Even when occupation was factored in as a covariate, no relationship between workplace conflict and depressive symptoms was evident, with variations in depressive symptoms among employees being more closely tied to individual factors than to the extent or frequency of workplace conflicts. While some individuals grapple with high-intensity workplace conflicts, they may employ positive coping mechanisms to address these conflicts, thereby averting detrimental effects on their physical and mental health (15, 79). After reviewing the studies, it has been determined that the objective environmental stimulus of workplace conflict does not directly contribute to employee depression. According to Transactional Stress Theory, both the environment and individuals play a role in affecting employees' physical and mental health through a complex interaction process. Individuals constantly evaluate stimuli in their environment, which triggers different coping styles to address stressors. Appropriate coping styles are beneficial for conflict resolution, while inappropriate ones may lead to physical and mental issues (16, 17). Conflict management style refers to an individual's preferred approach when dealing with conflicts (18–20). It reflects an individual's enduring coping strategy in conflict situations and is influenced by a combination of personality traits (21) and situational factors [such as cultural background—(22, 23)]. Therefore, compared to workplace conflict itself, an individual's management style toward conflict may be the underlying cause of employee depression.

### 1.1 Influence of conflict management style on depressive symptoms of employees

Research by Afzalur Rahim (82), and Thomas and Kilmann (24) identified five primary conflict management styles prevalent among Western employees: competition, collaboration, avoidance, compromise, and accommodation, leading to the development of a conflict management style scale based on these styles. In reality, individuals do not adopt a single conflict management behavior when faced with conflicts; rather, their actions are often a combination of multiple conflict management behaviors (25). Furthermore, under the influence of Confucian culture, the Chinese way of dealing with situations typically embodies collectivism and prioritizes harmony. Consequently, they are less likely to engage in direct confrontation or competition with others in conflict situations, and more likely to adopt conceding conflict management strategies (26). Consequently, Chen et al. (18) created a culturally adapted conflict management style scale integrating Chinese attributes and prior research. Subsequent to this, Liu et al. (27) further refined the scale in studies specifically related to workers. Drawing on the research findings of Chen et al. (28) and Wang and Jing (87), they categorized the conflict management styles of Chinese employees into two main types: coordination style and concession style. Specifically, the coordinating style is a blend of collaboration and compromise, while the conceding style is a combination of avoidance and accommodation (27). The coordination style entails employees seeking cooperation or finding a middle ground to achieve mutually beneficial outcomes when confronted with workplace conflicts. On the other hand, the concession style involves employees attempting to avoid, tolerate, and comply with conflicts in the workplace (29).

Previous research on conflict management styles has predominantly focused on their impact on work performance, team innovation, and overall productivity (30), as well as strategies for cultivating positive conflict management styles (31). However, there is a noticeable gap in studies directly comparing the effects of different conflict management styles on physical and mental health, particularly in relation to depression. For instance, Chung-Yan and Moeller (79) explored the impacts of an integrative and compromising conflict management style on social dysfunction and anxiety/depression. In a study involving Dutch workers, De Dreu et al. (15) discovered a correlation between avoidance and accommodation conflict management styles and increased physical discomfort, such as fatigue and headaches. Existing research has not definitively elucidated the effects of various conflict management styles on workers' depressive symptoms. The Transactional Stress Theory posits that the efficacy of conflict management strategies can impact an individual's physiological responses and long-term health status. Prolonged avoidance of conflicts may exacerbate health issues (16, 17). From the perspective of coping style, individuals with a coordination style may employ more effective conflict management strategies, while those with a concession style may exhibit contrasting outcomes. Consequently, these two types of conflict management styles are likely to have a closer association with the physical and mental wellbeing of employees. Previous studies on coping styles have shown that college students adopting positive coping styles can effectively reduce the risk of depression (32). It has been observed that adopting negative coping styles can heighten the risk of depression and anxiety among individuals (33–36). Zhao et al. (37) discovered a significant correlation between diverse coping styles employed in managing interpersonal conflicts within dormitories and the levels of depression experienced by college students. Specifically, negative styles such as competition and avoidance exhibited a noteworthy positive association with depression, while positive styles like cooperation demonstrated an opposing effect. Similar findings were reported in studies analyzing family conflict resolution styles. Choi et al. (38) highlighted that negative family conflict resolution approaches were more likely to lead to depressive symptoms compared to positive resolutions. Building upon these insights, hypothesis 1 posits that employees adopting a concession style in conflict situations are more prone to experiencing higher levels of depression compared to those employing a coordination style. The concession style exhibits a positive correlation with employees' depressive symptoms, while the coordination style demonstrates a negative correlation with such symptoms.

### 1.2 The potential mechanism by which conflict management style affects workers' depressive symptoms: the mediating role of exhaustion

The current understanding of the internal mechanism through which conflict management styles influence workers' depression is still not fully elucidated. However, drawing from the conservation of resources theory proposed by Hobfoll and Lerman (39), it is suggested that emotional exhaustion may play a pivotal role in this relationship. Emotional exhaustion is a persistent state of physical and emotional depletion resulting from factors like overwork or unmet personal needs (40, 83). The theory of resource conservation posits that individuals possess an inherent inclination to preserve, safeguard, and acquire valued resources. Consequently, the actual or potential loss (or threat) of resources can induce stress and tension in individuals. To avert the loss of a valued resource (e.g., job retention), individuals will allocate more of their own resources toward its protection. Nevertheless, due to individuals' limited pool of resources, prolonged utilization without sufficient replenishment can result in heightened levels of emotional exhaustion, which may manifest as adverse outcomes such as emotional breakdowns and diminished performance (41). The job demands-resources (JD-R) model proposes that working conditions can be categorized into two overarching domains: job demands and job resources. Job demands encompass the physical, social, or organizational aspects of a job that necessitate sustained physical or mental effort, primarily relating to the exhaustion component of burnout (42, 43). When an individual's resources are insufficient to meet the demands of their job, it can result in emotional exhaustion. The concession style, as compared to the coordination style, involves staying away from conflicts and demands a substantial investment of personal resources. For example, the individual with a concession style will exert greater effort to suppress their emotions and enhance work performance when faced with conflicts with a leader, in order to secure their job position. However, addressing conflicts solely through avoidance and personal resource investment without tackling the underlying issues could exacerbate conflicts and eventually deplete individuals' limited resources, as outlined by Baumeister et al. (44). Conversely, the coordination style represents a more constructive approach to conflict management (45), it can effectively resolve conflicts and mitigate resource depletion, eliminating the need for individuals to constantly deplete their own resources in order to preserve valuable ones (84). Research by García-Arroyo and Segovia (46) focusing on university teachers revealed a positive correlation between negative coping styles and emotional exhaustion, while positive coping styles exhibited a negative correlation with emotional exhaustion. Additionally, Smout et al. (47) found that the utilization of maladaptive coping styles was linked to increased emotional exhaustion. These findings suggest that employees adopting different conflict management styles may experience varying levels of emotional exhaustion.

Indeed, emotional exhaustion has been identified as a central component of job burnout (48), individuals with emotional exhaustion will have physical fatigue and a sense of feeling emotionally “drained,” the long-term consequences of this will have an impact on both physical and mental wellbeing. A recent meta-regression study which contained 69 studies based on 46,191 participants found that the mean correlation coefficient between emotional exhaustion and depression was 0.53, and the effect size was 0.55, which was a large effect size (85). And numerous studies have demonstrated its positive association with depression (49, 50). Research has consistently shown that emotional exhaustion in the workplace can serve as a precursor to depressive symptoms (49, 51, 52), leading to physical fatigue, hardly work, intentions to resign, and ultimately contribute to the development of depression (53). Therefore, Emotional exhaustion serves as a significant predictor of depression.

In summary, various conflict management styles can impact an individual's level of depression by altering emotional exhaustion levels. Therefore, hypothesis 2 of the study posits that emotional exhaustion acts as a mediating factor in the relationship between conciliation style, coordination style, and employee depression.

## 2 Methods

### 2.1 Subject

In this study, a total of 1,312 online questionnaires were gathered from employees in Tianjin using the Questionnaire Star platform (https://www.wjx.cn/). In order to assess the quality of respondents' answers, two validation questions are included in the questionnaire. For instance, please choose the “almost none” option provided here. Participants who fail to comply with this requirement will be considered invalid. After removing 285 invalid responses, 1,027 valid responses were retained, resulting in the efficient response rate of 78.28%.

The study conducted by Sato et al. (54) revealed a significant correlation between the temporal aspects of work and the mental wellbeing of employees, with prolonged working hours or overtime being associated with a decline in workers' mental health. According to Sambasivam et al. (55), there is a strong association between job categories and mood disorders, with Clerical support workers being more susceptible to developing mood disorders compared to enterprise technicians or managers. Therefore, the study also evaluated the participants' work-related requirements, including their job categories, average working hours, and overtime situation, in addition to demographic information such as age, gender, and education level. The Job demand-resource model posits that higher work demands entail longer durations and greater sustained physical or mental effort, which in turn leads to adverse health outcomes (56). According to statistics, the participants' ages ranged from 18 to 62 years, with an average age of 37.28 ± 8.43 and an average working time of 8.95 ± 1.76 h. Table 1 presents the demographic details of the survey respondents.

**Table 1:** Basic information of survey objects.

**Distribution characteristics**	**Number**	**Percent**
**Sex**		
Male	361	35.2%
Female	666	64.8%
**Educational level**		
Middle school or below	111	10.8%
High or vocational school	145	14.1%
Bachelor or the equivalent's	692	67.4%
Master or above	79	7.7%
**Overtime situation**		
Frequently	241	23.5%
Sometimes	631	61.4%
Never	155	15.1%
**Job category**		
Enterprise staff	543	52.9%
Enterprise management	205	20.0%
Government agency	279	27.2%

### 2.2 Tools

#### 2.2.1 Conflict management style questionnaire

In this study, the conflict management style questionnaire revised by Liu et al. (27) was utilized to assess employees' behavioral responses when confronted with conflict. The questionnaire comprises 15 items and is rated on a 5-point scale, where “1” indicates “completely disagree” and “5” signifies “completely agree.” The questionnaire encompasses two subscales: coordination style and concession style. The coordination style subscale corresponds to questions 1–7, the higher the score, the greater the inclination for individuals to engage in cooperative behavior during conflicts, aiming to achieve mutually beneficial outcomes. One example item includes, “I attempt to discuss problems with my colleagues and seek mutually acceptable solutions.” The concession style subscale corresponds to questions 8–15, the higher the score, the greater the likelihood that the individual will exhibit tolerance, avoidance, or compliance toward the conflicter (18, 27, 29). One example item includes, “I am inclined to be patient and obedient toward my colleagues.” In this study, the Cronbach's α coefficients for the two subscales were 0.97 and 0.90, respectively. With regards to evidence of construct validity we performed a confirmatory factor analysis (CFA) for each questionnaire which coordination style and concession style were taken as two latent factors. The χ^2^-value and fit indexes were $\chi_{\left(90\right)}^{2}$ = 614.90 (*p* < 0.001), CFI = 0.93, TLI = 0.92, RMSEA = 0.08, SRMR = 0.09, with standardized loadings ranging between 0.49 and 0.96. In summary, χ^2^-value is high, which is expected due to the large sample size and the simplicity of the model (81), but RMSEA, CFI and TLI values indicate the questionnaire is adequate.

#### 2.2.2 Patient Health Questionnaire Depression Scale

The Patient Health Questionnaire Depression Scale (PHQ-9) is a widely utilized tool for assessing depression or the severity of depression (57), and has been extensively employed across various professional domains such as healthcare practitioners (58), autoworkers (59). This scale comprises nine items designed to gauge participants' depressive moods over the preceding 2 weeks, with scores ranging on a 4-point scale, where “0” represents “not at all” and “3” signifies “almost every day.” One example item includes, “Feeling tired or lacking in energy.” The depression score is calculated as the aggregate of all questions, ranging from 0 to 27, with higher scores indicating greater levels of depression. Specific score ranges correspond to varying levels of depression severity: 0–4 indicates no depression symptoms, 5–9 suggests mild depression symptoms, 10–14 denotes moderate depression symptoms, and 15 or higher indicates severe depression symptoms (60). In this study, the Cronbach's α coefficient for this questionnaire was 0.91, signifying high internal consistency reliability. The fit indexes were $\chi_{\left(27\right)}^{2}$ = 269.83 (*p* < 0.001), CFI = 0.92, TLI = 0.89, RMSEA = 0.09, SRMR = 0.05, with standardized loadings ranging between 0.54 and 0.84. The RMSEA and TLI values are close to the standard values of 0.08 and 0.9, and CFI value is >0.9, which indicates the scale is adequate.

#### 2.2.3 Emotional exhaustion questionnaire

The emotional exhaustion subscale from the job burnout questionnaire developed by Li and Wu (61) was employed in this study to assess employees' levels of emotional exhaustion. This questionnaire, known for its high reliability and validity, has been widely applied in various professional domains such as education, law enforcement, management, and employee assessment. The emotional exhaustion subscale comprises five items, rated on a 7-point scale, where “1” indicates “completely inconsistent” and “7” represents “completely consistent.” Higher scores correspond to greater levels of emotional exhaustion. One example item includes, “I have been feeling exhausted frequently.” In the context of this study, the Cronbach's α coefficient for the emotional exhaustion dimension was calculated to be 0.88. The fit indexes of CFA were $\chi_{\left(5\right)}^{2}$ = 15.1 (*p* < 0.001), CFI = 1.00, TLI = 0.99, RMSEA = 0.04, SRMR = 0.02, with standardized loadings ranging between 0.69 and 0.86.

### 2.3 Data analysis

Firstly, we conducted descriptive statistics on employees' depression scores, and to investigate the levels of depression severity among employees, the depression score in this study was transformed into depression severity based on the criteria outlined in the PHQ for assessing depression severity (60). Then the present study employed ANOVA to examine the variations in depression scores across different worker groups, considering factors such as gender, education level, job category, and overtime situation. SPSS26.0 was utilized for data analysis.

Secondly, to investigate the influence of different conflict management styles on employee depression, the study computed the conflict management style preference score by subtracting the concession subscale score from the coordination subscale score. The dataset was then sorted based on this index, and the top 27% and bottom 27% of the data were selected to represent the high coordination style group and high concession style group, respectively. Subsequently, an independent samples *t*-test was performed on the depression scores of these two groups of participants, with a significance level set at 0.05 to determine any statistically significant differences.

Thirdly, The Maximum Likelihood estimation method within Mplus (v8.3) was employed to conduct the analysis of a structural equation model, focusing on employees' coordination style, concession style, depression scores, and emotional exhaustion scores. The bootstrap method was used to test the mediating effect (sample size 5,000, 95% confidence interval). Before proceeding with the analysis, the measurement models were initially subjected to testing. Then, the mean, standard deviation of the variables, and correlation coefficients between them were calculated. To examine the impact of each control variable on exhaustion and depression scores, we conducted a correlation analysis for continuous variables (age and average working hours). For categorical variables (job category, gender, level of education, and situation of overtime), analysis of variance was performed. The analyses were conducted utilizing SPSS 26.0. The structural equation model should incorporate control variables that have significant effects.

In the final stage of the study, In order to test the robustness of the model effects, PROCESS (V4.1) for SPSS 26.0 was utilized to conduct a mediation analysis. Model 4 was selected, with the subjects' conflict management styles (coordination style/concession style) as the independent variable (X), depression score as the dependent variable (Y), and emotional exhaustion score as the mediator (M), control variables with significant effects as covariates in the analysis. The bootstrap method was used to test the mediating effect [sample size 5,000, 95% confidence interval; (62)], if the confidence interval does not include 0, then the path is valid.

## 3 Results

### 3.1 Depression scores and group differences among workers

The depression score of the workers in this study was 5.21 (SD = 4.79). According to the statistics gathered, it was found that 52.2% of employees exhibited no depressive symptoms, 34.0% experienced mild depressive symptoms, 9.1% showed moderate depressive symptoms, and 4.8% displayed severe depressive symptoms. A score of 10 on the PHQ-9 has been identified as the optimal threshold value for demarcation (58, 60, 63), the prevalence of moderate to severe depression among the workers was 13.9%.

**Figure 1:**
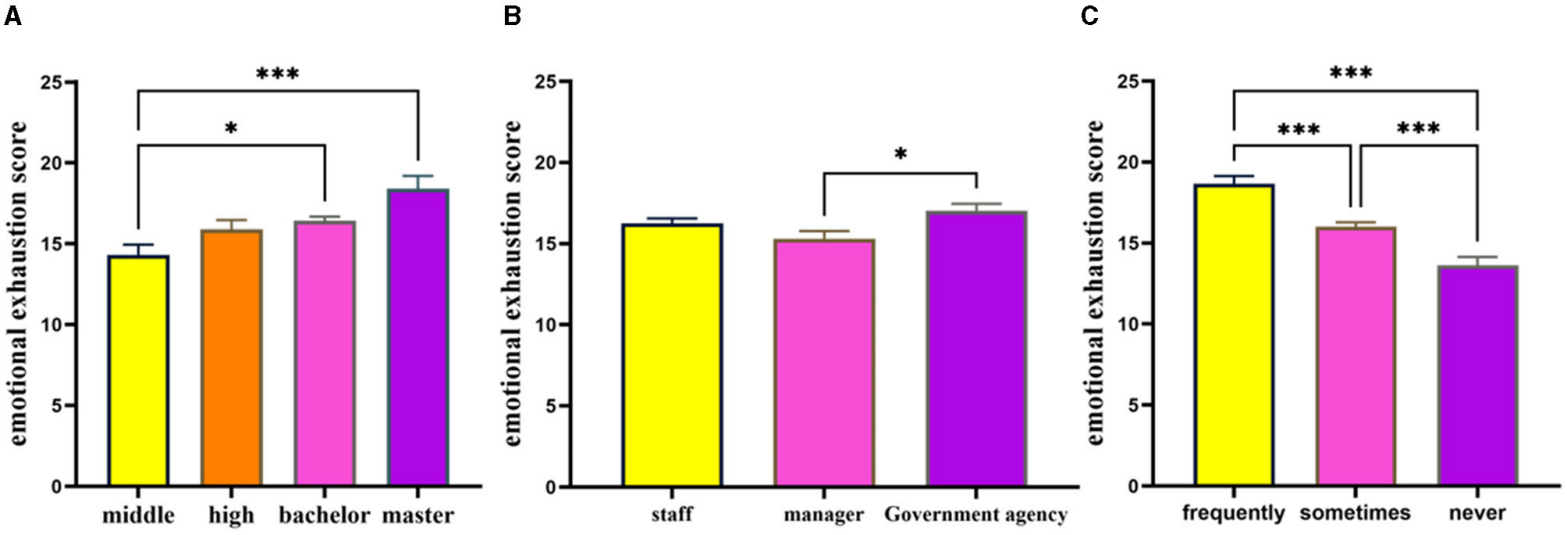
Group difference in depression scores of workers. The variables education level **(A)**, job category **(B)**, and overtime situation **(C)** all significant influences on the depression scores. ^***^Represents *p* < 0.001, ^**^represents *p* < 0.01, ^*^represents *p* < 0.05.

The analysis revealed no significant difference between gender and the depression score. The depression scores were found to be significantly impacted by factors such as educational level [*F*_(3, 1, 023)_ = 5.42, *p* = 0.001, partial η^2^ = 0.016, Middle school or below < Bachelor or above], job category [*F*_(2, 515.12)_ = 12.21, *p* < 0.001, partial η^2^ = 0.021, enterprise management < government agency], and situation of overtime work [*F*_(2, 347.86)_ = 14.58, *p* < 0.001, partial η^2^ = 0.033 Never < Sometimes < Frequently] as illustrated in Figure 1. The job category and overtime situation fail to meet the homogeneity test of variance, thus these two reports are the results of welch test. Cohen (64) provided guidelines for the interpretation of partial η^2^ and Cohen's *d*: values of 0.01, 0.06, 0.14 for partial η^2^ and values of ±0.20, ±0.50, ±0.80 for Cohen's *d* are commonly considered to be indicative of small, medium, and large effects. The effect sizes of the aforementioned findings are therefore deemed to be small.

### 3.2 Influence of conflict management style on depressive scores of employees

**Figure 2:**
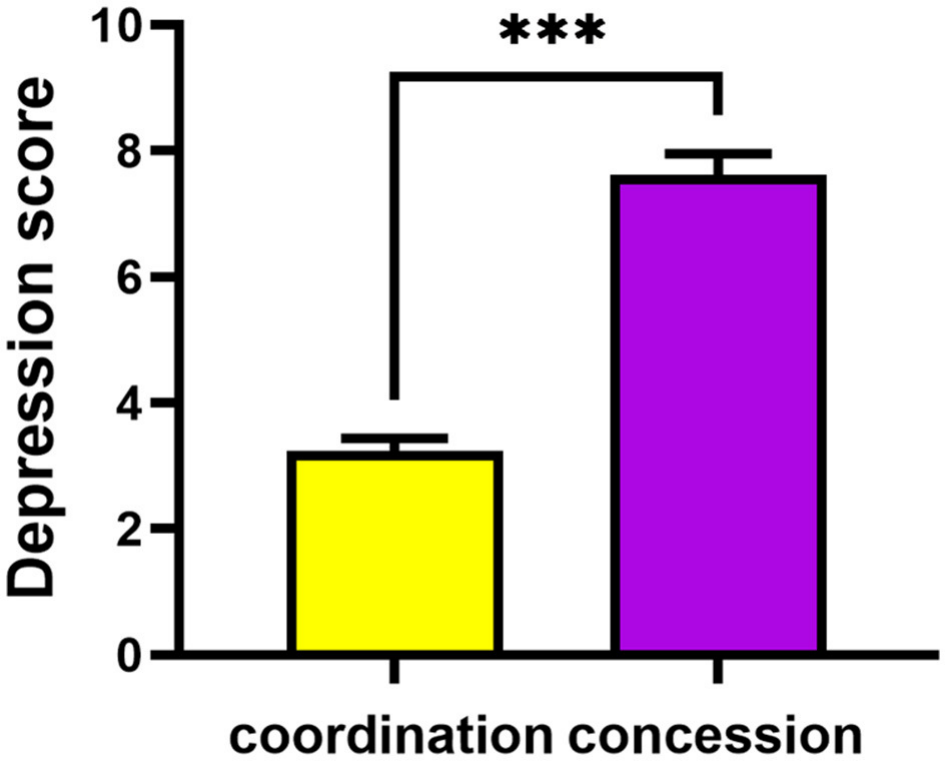
Depression scores of the high coordination style group and the high conciliation style group. The depression score of the high coordination style group exhibited a significantly greater magnitude compared to that of the high concession style group. ^***^Represents *p* < 0.001, ^**^represents *p* < 0.01, ^*^represents *p* < 0.05.

The independent samples *t*-test was carried out to compare the depression scores between the high coordination style group and the high concession style group. The results revealed a significant difference, indicating that the depression score of the high coordination style group was notably lower than that of the high concession style group [*t*_(454.62)_ = −11.46, *p* < 0.001, Cohen's *d* = −0.973], as depicted in Figure 2. This suggests that employees who exhibit a preference for concession in conflict situations tend to have higher depression scores compared to those who lean toward a coordination style of conflict management. The effect size is substantial and the results are robust.

### 3.3 The mediating role of emotional exhaustion

#### 3.3.1 Tests of measurement models

To assess the validity of our proposed measurement model, we performed a series of CFAs encompassing all multi-item constructs, i.e., coordination style (seven-items), concession style (eight-items), depression (nine-items), emotional exhaustion (five-items). It is important to note that larger CFI and TLI values, as well as smaller RMSEA and SRMR values, indicate better models. As shown in Table 2, a CFA of four-factor model produced a good fit: $\chi_{\left(371\right)}^{2}$ = 2,230.57 (*p* < 0.001), CFI = 0.92, TLI = 0.91, RMSEA = 0.07, SRMR = 0.06, the details in Table 3. Our hypothesized model produced significantly better fit than the next best-competing model (Δχ^2^ = 790.21, *p* < 0.001) in which we loaded the items of emotional exhaustion and depression on one signal latent factor: $\chi_{\left(374\right)}^{2}$ = 3,020.78 (*p* < 0.001), CFI = 0.89, TLI = 0.88, RMSEA = 0.08, SRMR = 0.07.

**Table 2:** Comparisons of measurement models using confirmatory factor analysis.

**Measurement models**	***χ^2^***	***p***	***df***	****Δχ* ^2^***	**CFI**	**TLI**	**RMSEA**	**SRMR**
Hypothesized four-factor model	2,230.57	< 0.001	371	—	0.92	0.91	0.07	0.06
**Alternative models**								
1. Three-factor model with coordination style and concession style combined	6,249.88	< 0.001	374	4,019.31	0.75	0.73	0.12	0.15
2. Three-factor model with coordination style and emotional exhaustion combined	4,939.46	< 0.001	374	2,708.89	0.81	0.79	0.11	0.15
3. Three-factor model with coordination style and depression combined	6,947.32	< 0.001	374	4,716.75	0.72	0.70	0.13	0.18
4. Three-factor model with concession style and emotional exhaustion combined	5,424.55	< 0.001	374	3,193.98	0.78	0.77	0.12	0.18
5. Three-factor model with concession style and depression combined	7,039.54	< 0.001	374	4,808.97	0.72	0.69	0.13	0.19
6. Three-factor model with emotional exhaustion and depression combined	3,020.78	< 0.001	374	790.21	0.89	0.88	0.08	0.07
7. One-factor model with all latent variables combined	13,027.22	< 0.001	377	10,796.65	0.46	0.42	0.18	0.23

N = 1,027.

CFI, Comparative Fit Index; TLI, Tucker-Lewis Index; RMSEA, Root Mean Square Error of Approximation; SRMR, Standardized Root Mean Square Residual.

**Table 3:** Results of confirmatory factor analyses of the hypothesized four-factor model (*N* = 1,027).

**Item**	**Factors**				**S.E**.	***Z***	***P*-value**
	**Coordination style**	**Concession style**	**Emotional exhaustion**	**Depression**			
I make an effort to have discussions with my colleagues about problems and find mutually acceptable solutions.	0.921				0.005	177.366	^***^
I strive to share precise information with my colleagues and collaborate in problem-solving.	0.956				0.003	293.659	^***^
I make an effort to collaborate with my colleagues in order to reach a correct understanding of the problem.	0.961				0.003	323.051	^***^
I make an effort to combine my ideas with those of my colleagues and strive to find common ground.	0.920				0.005	176.541	^***^
I collaborate with my colleagues to find mutually satisfactory solutions.	0.910				0.006	155.963	^***^
I endeavor to identify a compromise solution to overcome the deadlock.	0.856				0.009	96.896	^***^
I make an effort to engage in negotiations with my colleagues in order to find a mutually agreeable solution.	0.804				0.011	69.923	^***^
I frequently make concessions to my colleagues.		0.577			0.022	25.784	^***^
I am inclined to be patient and obedient toward my colleagues.		0.805			0.013	62.606	^***^
I usually prefer to remain silent.		0.768			0.015	52.619	^***^
I chose to back down and avoid arguing with my colleagues.		0.830			0.012	71.123	^***^
I try to avoid awkward situations and refrain from discussing conflicts between my colleagues and me.		0.722			0.017	43.559	^***^
I am inclined to defer to the wishes of my colleagues.		0.822			0.012	67.545	^***^
I have a tendency to choose avoidance.		0.753			0.015	49.214	^***^
I usually follow the suggestions of my colleagues.		0.485			0.025	19.063	^***^
I am experiencing significant fatigue.			0.846		0.011	75.964	^***^
I am concerned that work may have an impact on my emotional state.			0.686		0.018	37.515	^***^
I frequently feel exhausted.			0.863		0.010	82.489	^***^
After a day's work, I experience profound fatigue.			0.738		0.016	45.973	^***^
Recently, I have been experiencing a sense of melancholy.			0.727		0.017	43.433	^***^
Little interest or pleasure in doing things.				0.800	0.013	62.101	^***^
Feeling down, depressed or hopeless.				0.836	0.011	75.242	^***^
Trouble falling asleep, staying asleep, or sleeping too much.				0.686	0.018	38.244	^***^
Feeling tired or having little energy.				0.770	0.014	53.939	^***^
Poor appetite or overeating.				0.681	0.018	37.472	^***^
Feeling bad about yourself or that you're a failure or have let yourself or your family down.				0.778	0.014	56.045	^***^
Trouble concentrating on things, such as reading the newspaper or watching television.				0.690	0.018	38.756	^***^
Moving or speaking so slowly that other people could have noticed. Or, the opposite being so fidgety or restless that you have been moving around a lot more than usual.				0.710	0.017	41.720	^***^
Thoughts that you would be better off dead or of hurting yourself in some way				0.541	0.023	23.112	^***^

^***^Represents *p* < 0.001, ^**^represents *p* < 0.01, ^*^represents *p* < 0.05, the same applies thereafter.

To examine the discriminant validity of the latent constructs, we assess the average variance extracted (AVE) for each factor in our hypothesized measurement model. The AVE for coordination style, conciliation style, emotional exhaustion, and depression were 0.82, 0.53, 0.60, and 0.53, respectively; all of which exceeded the threshold of 0.5 (65).

#### 3.3.2 Descriptive statistics and control variables assessment

The descriptive statistics and correlation analysis were conducted on the scores of coordination style, concession style, emotional exhaustion, depression, age and average working hours among the survey participants. The results are presented in Table 4. It was found that there was no significant correlation between coordination style and concession style. However, a significant positive correlation was observed between emotional exhaustion and depression. Additionally, there was a significant negative correlation between coordination style and both emotional exhaustion and depression, and concession style exhibited a significant positive correlation with both emotional exhaustion and depression. The results also indicated a negative correlation between age and depression, as well as a positive correlation between average working hours and both exhaustion and depression.

**Table 4:** Descriptive statistics and correlation analysis matrix of variables.

**Variable**	**Average**	**SD**	**1**	**2**	**3**	**4**	**5**
1. Emotional exhaustion	16.27	7.05	—				
2. Coordination style	27.97	3.91	−0.40^***^	—			
3. Concession style	25.31	5.03	0.16^***^	0.02	—		
4. Depression	5.21	4.79	0.70^***^	−0.30^***^	0.21^***^	—	
5. Age	37.28	8.43	−0.05	0.03	−0.05	−0.10^**^	—
6. Average working time	8.96	1.76	0.19^**^	−0.05	0.06	0.14^**^	−0.04

^***^Represents *p* < 0.001, ^**^represents *p* < 0.01, ^*^represents *p* < 0.05.

**Figure 3:**
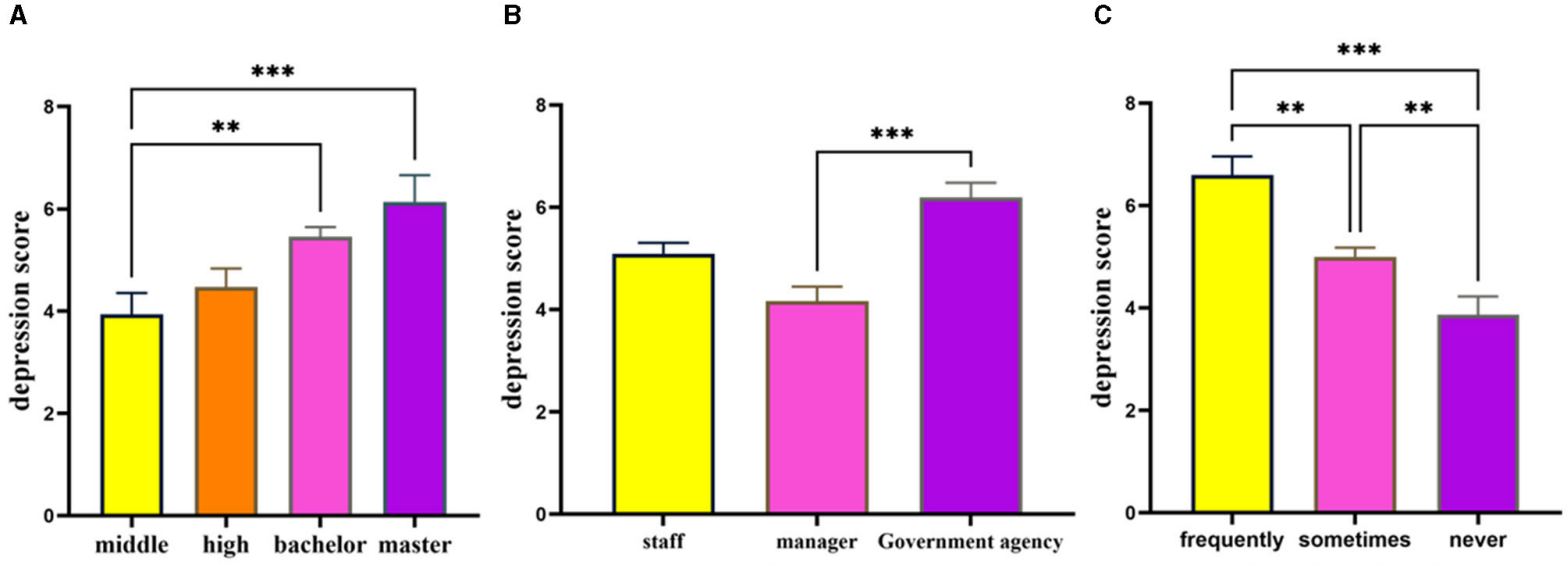
Group difference in emotional exhaustion of workers. The variables education level **(A)**, job category **(B)**, and overtime situation **(C)** all significant influences on the emotional exhaustion. ^***^Represents *p* < 0.001, ^**^represents *p* < 0.01, ^*^represents *p* < 0.05.

The results of the control variable assessment indicated that emotional exhaustion was significantly influenced by education level [*F*_(3, 1, 023)_ = 5.63, *p* = 0.001, $\eta_{p}^{2}$ = 0.016, Middle school or below < Bachelor or above], job category [*F*_(2, 1, 024)_ = 3.50, *p* = 0.031, $\eta_{p}^{2}$ = 0.007, enterprise management < government agency], and overtime situation [*F*_(2, 1, 024)_ = 26.43, *p* < 0.001, $\eta_{p}^{2}$ = 0.049, Never < Sometimes < Frequently], while gender did not have a significant effect [*F*_(1, 1, 025)_ = 1.79, *p* > 0.05], details in Figure 3. And the findings in Part 3.1 indicate that depression score are influenced by factors such as education level, job category, and situation of overtime work, while gender does not play a significant role.

In summary, all control variables had a significant impact, with the exception of gender, which exhibited no influence on exhaustion and depression scores, as well as age, which demonstrated no effect on exhaustion scores. The aforementioned variables are thus incorporated as covariates in subsequent analyses.

#### 3.3.3 Structural equation model analysis

**Figure 4:**
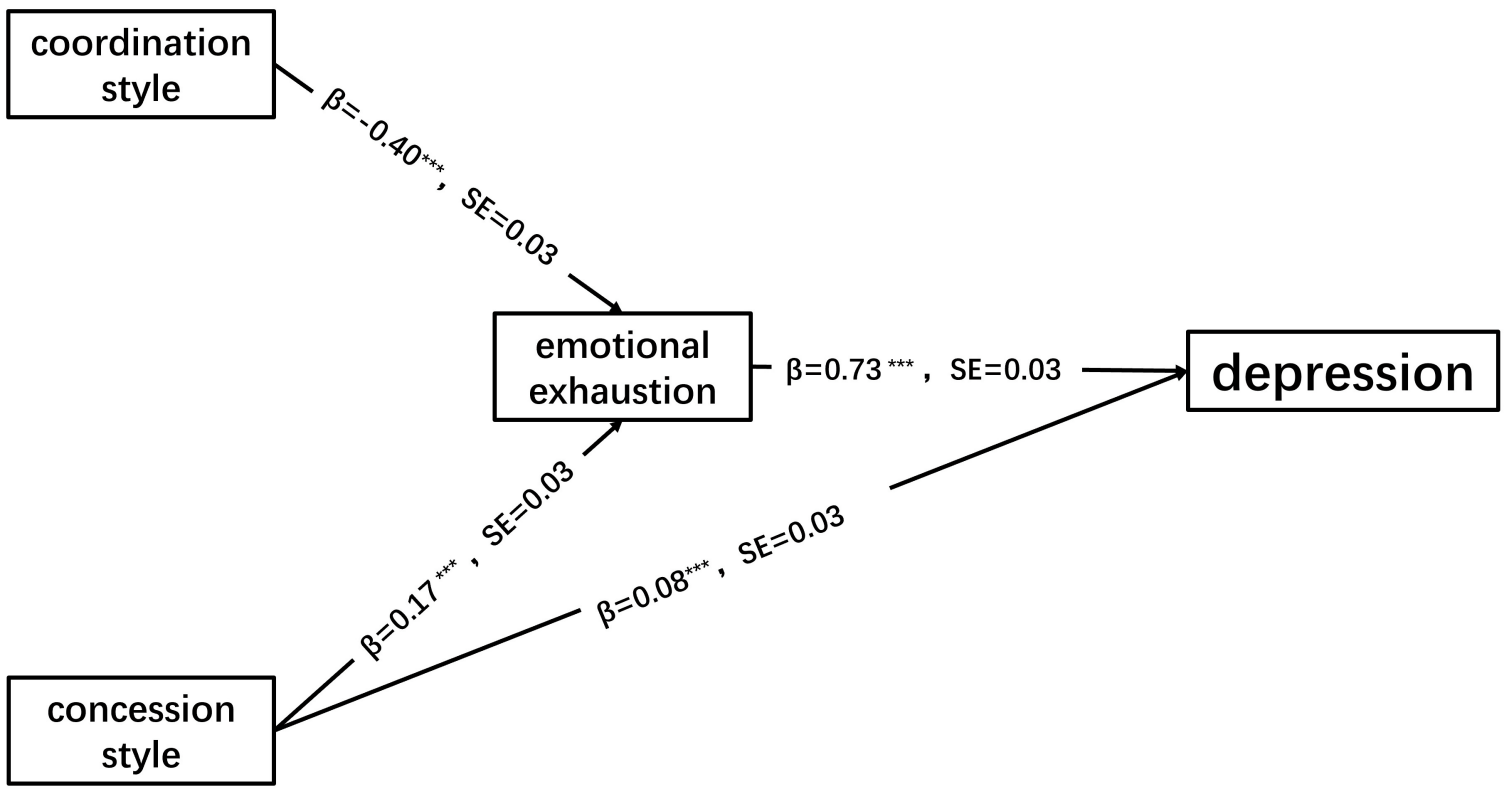
The mediating role of emotional exhaustion in the impact of different conflict management styles on depression. ^***^Represents *p* < 0.001, ^**^represents *p* < 0.01, ^*^represents *p* < 0.05.

The structural equation model was employed to investigate the mechanism by which coordination style and concessional style impact depression, with exhaustion serving as the mediating variable, education level, job category, overtime situation, and working hours acting as controlling variables for exhaustion and depression, and age acting as a controlling variable for depression. The model demonstrates good fit based on the following fitting indices: $\chi_{\left(616\right)}^{2}$ = 2,630.98 (*p* < 0.001), CFI = 0.92, TLI = 0.91, RMSEA = 0.06, SRMR = 0.06, as shown in Figure 4 (to simplify the model, control variables are omitted and only significant paths are presented). According to Figure 3, the coordination style demonstrates a significant negative association with exhaustion (β = −0.40, *p* < 0.001). The concessional style exhibits significant positive associations with exhaustion (β = 0.17, *p* < 0.001) and depression (β = 0.08, *p* = 0.001). Exhaustion significantly predicts depression (β = 0.73, *p* < 0.001). The mediating path was examined using a bias corrected bootstrap test. Findings revealed that the direct impact of coordination style on depression did not reach statistical significance [β = −0.002, 95% CI (−0.07, 0.06)]. However, there was a significant mediating effect of coordination style on depression through exhaustion [β = −0.30, 95% CI (−0.35, −0.24)]. Furthermore, the direct influence of concessional style on depression was found to be significant [β = 0.08, 95% CI (0.04, 0.14)], and the mediating effect of concessional style on depression through exhaustion was also significant [β = 0.13, 95% CI (0.08, 0.18)].

#### 3.3.4 Mediation analysis

A simple mediation test was conducted for coordination style-emotional exhaustion-depression and concession style-emotional exhaustion-depression, respectively. The covariates included age, level of education, job category, situation of overtime, and average working hours. The results of Bootstrap analysis showed that in the mediation test of coordination style-emotional exhaust-depression, coordination style had no significant direct Effect on depression [*Effect* = −0.002, *SE* = 0.03, 95% CI (−0.07, 0.06)]. Emotional exhaustion played a complete mediating role between coordination style and depression [*Effect* = −0.30, *SE* = 0.03, 95% CI (−0.35, −0.25)]. In the mediating test of concession style-emotional exhaust-depression, concession style had a significant direct effect on depression [*Effect* = 0.09, *SE* = 0.03, 95% CI (0.05, 0.14)]. Emotional exhaustion played a partial mediating role between concession style and depression [*Effect* = 0.12, *SE* = 0.03, 95% CI (0.07, 0.17)].

## 4 Discussion

The aim of this study was to examine the relationship between employees' conflict management styles, emotional exhaustion, and depressive symptoms in order to gain insight into the current prevalence of depression among employees and explore how different conflict management styles influence employee depression and its underlying processes. Results revealed that the prevalence of moderate to severe depression among the workers was 13.9%. Specifically, utilizing a coordination style for conflict management was associated with lower depression scores, whereas employing a concession style was linked to higher depression scores, with emotional exhaustion acting as a mediating factor in these relationships.

### 4.1 The current situation regarding employee depression

According to the findings of this study, 52.2% of employees did not exhibit any depressive symptoms, while 34.0% displayed mild depressive symptoms, 9.1% demonstrated moderate symptoms, and 4.8% experienced severe symptoms of depression. The data indicates that a total of 47.8% of employees showed varying degrees of depression, with a predominant focus on mild depressive symptoms. Setting the cutoff for PHQ-9 at 10 would imply that at least 13.9% of workers would require attention (63). Furthermore, the present study revealed that demographic factors, including age, educational attainment, and job demand characteristics such as job category, situation of overtime work, and average daily working hours significantly influenced employees' levels of depression.

The present study revealed a weak negative correlation between age and employees' depression scores, while the depression scores increased with higher levels of education. The depression scores of workers with bachelor's and graduate degrees were found to be higher compared to those with middle school degrees. The findings in this study diverge from the conclusions drawn by previous studies. Firstly, Gao et al. (66) discovered a positive non-linear correlation between age and depression, indicating that as individuals grow older, their risk of experiencing depression increases. Further analysis revealed that the average age of the three respondent groups in the Gao's study was between 56 and 57 years old, with a standard deviation of ~7–8 years. The depression scores exhibited a consistent linear increase from ages 56 to 84 (mean~mean+3SD). Between the ages of ~32 and 56 (mean-3SD to mean), there was relatively little variation in depression scores, showing a slight negative trend (66). In this particular study, the participants had a mean age of 37.28 ± 8.43, and a weak negative correlation between age and depression scores was also observed. Secondly, previous studies have indicated a negative correlation between educational level and depression (67, 68). However, our study reveals an opposite trend. It is plausible that the job demands experienced by individuals with varying levels of education may contribute to this discrepancy. For instance, our findings demonstrate a significant association between educational attainment and job category. Specifically, individuals with higher education tend to be concentrated in government agencies, while those with lower education are more prevalent in enterprises staffs (as illustrated in Table 5).

**Table 5:** Relationship between cultural level and job position categories.

**Job category**	**Educational level (%)**				*** $\chi_{\left(df\right)}^{2}$ ***	**Cramer's *V***
	**Middle school or below**	**High or vocational school**	**Bachelor or the equivalent's**	**Master or above**		
Enterprise staff	18.6%	23.0%	54.0%	4.4%	193.33 (6)^***^	0.307
Enterprise management	4.4%	6.3%	83.4%	5.9%		
Government agency	0.0%	2.9%	81.7%	15.4%		

^***^Represents *p* < 0.001, ^**^represents *p* < 0.01, ^*^represents *p* < 0.05.

Consistent with previous research findings, job demand characteristics such as job category, average working hours, and situation of overtime have been found to exert an impact on employee depression. Specifically, government agencies exhibited significantly higher levels of depression compared to enterprise managers. Moreover, a positive correlation was observed between longer working hours and elevated depression scores, while increased situation of overtime was associated with heightened levels of depressive symptoms. Existing studies have found that job categories affect workers' physical and mental health (69–71). For example, direct exposure to the public, exposure to disease, occupations involving physical labor, and working with people are risk factors for depression, while leisure time, communication, decision-making, creativity, reasoning, responsibility at work, etc., are protective factors for depression (69). Most of the government agencies included in this study were from the Healthcare Commission, Emergency Management Agency, and Education Bureau. Given their frequent exposure to diseases, stressful events, and public interactions as part of their job responsibilities, it is not surprising that they reported the highest levels of depression. On the other hand, enterprise managers who engage in decision-making processes, reasoning activities, and creative work reported comparatively lower levels of depression. It is worth noting that average working hours and situation of overtime serve as reliable indicators for assessing an individual's workload. A meta-analysis by Virtanen et al. (72) found a significant positive correlation between long working hours and depressive symptoms in Asia. Long working hours can positively predict the risk of mental illness (73) and have adverse effects on the health of workers, such as increasing the risk of musculoskeletal disease, cardiovascular disease, stroke, sleep disorders, etc. (74–76). The findings presented here provide further support for the Job Demand-Resource model, indicating that when job demands are excessively high, individuals tend to deplete their personal resources, ultimately impacting their physical and mental wellbeing and leading to depressive symptoms (56).

### 4.2 Conflict management style and depression

Previous studies have primarily focused on examining the direct link between workplace conflict and employee mental wellbeing. However, while certain research has shown no clear association between employee depression and workplace conflict (14), other studies have indicated a correlation between employee depression and their individual approaches to managing conflict (79). Building upon this foundation, the current study delved deeper into comparing the impacts of various conflict management styles on employee depression. The findings revealed that employees who utilize a coordination style in handling conflicts tend to exhibit lower levels of depression compared to those employing a concession style. Furthermore, it was observed that higher scores in the coordination style were linked to lower depression scores, whereas higher scores in the concession style were associated with elevated depression scores. These results substantiate hypothesis 1.

The findings also provide support for the Transactional Stress Theory, which posits that an individual's ability to effectively manage conflict has a direct impact on their physiological response and long-term health. Employees with a concession style tend to adopt handling methods such as avoidance, tolerance, and compliance when facing workplace conflicts. Rahim and Magner (86) view avoidance as a “lose-lose” conflict management approach. It fails to address issues fundamentally and merely involves advising oneself to concede and compromise with others, representing a passive way of coping. Numerous studies have shown that this passive coping style not only leads to physical discomfort but also has negative impacts on mental and physical health (15, 34, 36). In contrast, employees utilizing a coordinating style typically seek cooperation or find a middle ground for a win-win outcome when confronted with workplace conflicts. This problem-focused, constructive, and proactive approach allows individuals to actively address and resolve conflicts, eliminating sources of stress and effectively reducing the risk of depression (32).

### 4.3 Effect of emotional exhaustion

The emotional exhaustion scores of workers were also influenced by factors such as educational level, job category, overtime situation, and average working hours. According to the job demand-resource model (42, 56), individuals with higher job demands tend to consume more resources, leading to a perceived depletion effect. After controlling for the influence of these variables on emotional exhaustion and the influence of both these variables and age variable on depression, a structural equation model was established to examine the relationship between conflict management style, emotional exhaustion, and depression.

This study additionally uncovered that emotional exhaustion served as a mediating factor in the impact of both coordination style and concession style on employee depression, shedding light on the internal mechanisms through which different conflict management styles influence employee depression. Specifically, the coordination style primarily mitigates employees' depressive symptoms by diminishing emotional exhaustion. Conversely, the concession style not only heightens the level of depression by amplifying emotional exhaustion but also exerts a direct influence on employee depression. These findings align with the conservation of resources theory and the Job Demand-Resource model (39, 41, 42). The depletion of personal resources, whether due to work demands or the safeguarding of valuable assets, can occur as individuals strive to protect core resources or fulfill work-related needs. However, if these personal resources are not replenished in a timely manner, they will eventually be exhausted and may lead to depressive symptoms. The coordination style is considered a positive conflict management approach (45). This style involves individuals actively addressing and resolving conflicts to prevent the continuous depletion of their own resources (84). Employees who favor this conflict management style experience lower levels of emotional exhaustion, which aligns with findings from previous research studies (46). On the contrary, individuals who prefer the concessional style typically respond negatively to conflicts, often resorting to avoidance and obedience (44). However, this approach often comes at the expense of constant self-consumption (47). The higher the level of exhaustion, the greater the likelihood of experiencing depression, whereas lower levels of exhaustion are associated with reduced risk of depression (49, 51). Therefore, the coordinating style reduces the risk of depression by avoiding emotional exhaustion, while the concessional style accelerates resource depletion and promotes the development of depression.

The direct impact of the concession style on employee depression can be elucidated by the cognitive evaluation theory of emotion. According to this theory, when individuals employ emotion-centered negative coping styles (such as avoidance) in response to stressful events, they impede the development of adaptive health behaviors and consequently influence their physical and mental wellbeing (77). A substantial body of research has also indicated that negative coping methods can positively predict an individual's risk of depression (36, 37). However, the direct effect of the coordination style on employee depression was found to be insignificant, indicating that it does not exert a direct influence on employees' depressive symptoms. This demonstrates that different conflict management styles have varying effects on the internal processes related to employee depression.

### 4.4 Limitations and future directions

Firstly, Although this cross-sectional study examined employees in various positions, it is important to note that the findings do not establish causality, highlighting the need for subsequent longitudinal investigations to explore the causal mechanisms among variables.

Then, all measurement tools utilized in this study were self-report questionnaires. Despite the inclusion of detection questions, it was not possible to completely eliminate potential response biases exhibited by the participants, such as response bias, self-bias, and social desirability bias. Additionally, certain items within these questionnaires exhibited low factor loading; however, in accordance with the standardized scoring criteria for each scale, this study refrained from eliminating items with low factor loading. Upon examination, although the AVE of the factors measured by these scales exceeds 0.5, it is still likely to impact the measurement results. The future research should therefore focus on developing more efficient measurement tools and integrating additional data sources (such as observation, experimentation, or third-party reports) to enhance the validity of the findings.

Finally, drawing upon the Transactional Stress Theory and considering the conflict management preferences of Chinese workers, this study aims to investigate the association between coordination and concession as two conflict management styles and depression. The findings can be generalized to individuals with comparable cultural backgrounds. Future research could further explore how employee toward alternative conflict management styles relates to depression within diverse cultural contexts.

## 5 Conclusions

This study delved into the prevalence of depression among Chinese workers through online research and examined the relationships between various conflict management styles and workers' depression, as well as the mechanism of emotional exhaustion through a structural equation model. The findings indicate that (1) the prevalence of moderate to severe depression among the workers was 13.9%, highlighting the urgent necessity for immediate intervention in addressing the mental health concerns within the workforce. (2) Employees with different conflict management styles demonstrated varying levels of depression. Specifically, the high coordination style group exhibited significantly lower depression scores compared to the high concession style group. (3) Emotional exhaustion acts as a mediating factor in the relationship between both coordination style and concession style, and employee depression. Specifically, coordination style primarily alleviates employees' depressive symptoms by mitigating emotional exhaustion. Conversely, concession style not only directly affects employees' levels of depression but also exacerbates it by intensifying emotional exhaustion. The findings not only contribute to unveiling the internal mechanisms underlying employee depression in the workplace, thereby enriching research in related domains but also hold significant implications for the prevention and intervention of employee depression.

First and foremost, it is essential to provide comprehensive and easily accessible mental health services for employees. According to this survey, approximately half of the employees exhibit varying degrees of depressive symptoms, with 13.9% experiencing moderate to severe depression, warranting special attention. It is evident that depression issues are prevalent among the workforce. Therefore, it is imperative for companies to urgently establish mental health platforms for employees, ensuring that mental health services go deep into every workplace and every employee's family, enhancing service accessibility, and elevating the mental wellbeing of employees.

Secondly, it is essential to emphasize the promotion and mastery of psychological skills, such as adopting a positive conflict management approach. Companies should organize, support, and encourage departments to conduct ongoing psychological skills training programs. Through lectures, popular science education, group counseling, case studies, and various other forms, employees can be equipped with active conflict resolution skills and stress coping methods. By enhancing personal capabilities, individuals can protect their mental and physical wellbeing even in situations where the environment cannot be changed.

## Data Availability

The raw data supporting the conclusions of this article will be made available by the authors, without undue reservation.
